# Modulation in the elastic properties of gastrocnemius muscle heads in individuals with plantar fasciitis and its relationship with pain

**DOI:** 10.1038/s41598-020-59715-8

**Published:** 2020-02-17

**Authors:** Ji-Ping Zhou, Jia-Feng Yu, Ya-Nan Feng, Chun-Long Liu, Pan Su, Su-Hong Shen, Zhi-Jie Zhang

**Affiliations:** 1 Clinical Medical College of Acupuncture, Moxibustion and Rehabilitation, Guangzhou University of Chinese Medicine, Guangzhou, China; 2 Department of Rehabilitation Medicine, The First Affiliated Hospital of Zhengzhou University, Zhengzhou, China; 3 Luoyang Orthopedics Hospital of Henan Province, Luoyang, China

**Keywords:** Biotechnology, Risk factors

## Abstract

The objectives of this study were (1) to investigate the passive stiffness of the medial gastrocnemius (MG) and lateral gastrocnemius (LG) in patients with and without plantar fasciitis (PF), (2) to explore the correlation between gastrocnemius stiffness and plantar fascia thickness (PFT) as well as the intensity of pain in patients with PF, (3) to detect optimal cut-off points for stiffness of the MG and LG for identifying patients with PF. Forty *patients* (mean age = *51.1 years* ± *12.9) participated in* this study. The elastic properties of the MG and LG were quantified using shear wave elastography ultrasound. The thickness of the plantar fascia was measured *by B-mode imaging*. The intensity of pain was assessed using a visual analogue scale. The results showed that when the ankle was in the relaxed position, patients with PF had increased passive stiffness in the MG (*P* < 0.05) but not in the LG. Significant correlations were found between pain and the stiffness of the MG (middle, distal; all *P-values* < *0.05) and* no correlation was observed between pain and PFT (*P* = 0.416). The *initial* cut-off point for the stiffness of the MG was 29.08 kPa when the ankle was in the relaxed position. The findings from the present study show that an increase in muscle stiffness is not the same in the individual muscles of the gastrocnemius muscle. Traditional treatment of the whole gastrocnemius muscle might not be targeted at the tight muscle.

## Introduction

Plantar fasciitis (PF), which has a lifetime prevalence of approximately 10% in the general population, is most common among patients aged 40 to 60 years^1^. Patients with PF usually describe medial plantar heel pain on ambulation, especially during the first steps in the *morning, which* improves with weight bearing^2^. Biomechanical abnormalities are considered to be one of the main causes of PF and the risk factors for PF are multifactorial. These include reductions in the strength of calf muscles, tightness of the Achilles tendon (AT) and plantar fascia, isolated gastrocnemius tightness and abnormal foot alignment^3^. Overloading of the plantar fascia can lead to chronic degenerative *changes with* marked fibrosis and thickening within the fascia^4^. Anatomical studies describe the plantar fascia as connected to the AT, though *the AT consists of the gastrocnemius tendon and the soleus muscle tendon*. As such, there is strong evidence supporting the association of the gastrocnemius and the plantar fascia. However, the biomechanical effects of the gastrocnemius on the plantar fascia have not been fully addressed.

*Various calf muscles and fascia* (*e.g., the medial head of the gastrocnemius muscle MG] and the lateral head of the gastrocnemius muscle [LG])* are not only responsible for the dynamic stability of the ankle joint but also promote ankle plantar flexion and forward movement. *Among these functions, the elastic properties of the muscle are directly related to the contraction velocity and force production of muscles. Modulation in the elastic properties of muscle can reflect a pathological change and the recovery effect*^5^. The plantar fascia, the major arch-supporting structure of the foot, sustains high levels of stiffness during jumping and running. Previous studies have demonstrated that the gastrocnemius muscle is connected to the plantar fascia by means of the *AT*^6,7^. *Some* researchers have also shown that overstretching of the AT, which results from intense muscle contraction, is a plausible mechanical factor for excessive weight-bearing by the plantar fascia.

In a *cadaver experiment*, the strain on the plantar fascia increased with an increase in calf muscle tension^8^. Therefore, the excessive force produced by the MG and LG may alter the normal kinematics of ankle motion and therefore contribute to PF. However, the studies described above are based on a mechanical model of the foot and ankle or on cadaver observations. Although these methods can provide important information about the *MG, LG and plantar fascia*, they do not directly quantify muscle stiffness. Recently, most studies have demonstrated that reduced stiffness of the gastrocnemius muscle can alleviate the symptoms of patients with PF and can be achieved by activities such as stretching, massage, specific soft-tissue mobilization, and myofascial release^2,6,9,10^. In addition, our *recent study* demonstrated that an increase in muscle stiffness was not the same for the individual muscles of the quadriceps in patients with patellar tendinopathy (PT)^11^. Greater passive stiffness in the *vastus lateralis* (VL) was associated with higher patellar tendon stiffness^11^. As described, it is plausible that the passive stiffness of the muscle could vary in patients with and without PF. Furthermore, previous studies have evaluated muscle stiffness by examining the stiffness of individual regions of muscles (e.g., muscle belly)^7,11,12^. However, the stiffness in individual regions of *muscles* does not completely represent the stiffness change in the whole muscle because it is related to the viscoelastic properties of the whole muscle. Therefore, it is necessary that the values of the shear modulus of the MG and LG *are measured in* different muscle regions. *In summary, a more direct and accurate estimate of the biomechanical behavior of the gastrocnemius at different regions in patients with and without PF is important. It can provide a more comprehensive understanding of the physiological characteristics and pathological characteristics of muscles. Additionally, such measurements will lay a foundation for developing individualized and innovative preventive/rehabilitation strategies for patients with PF*.

Shear wave elastography (SWE) is an ultrasonographic imaging technique that allows noninvasive estimation of the elastic properties of the muscle^7,12,13^. *It is based* on the *fact, that* the elastic properties *are measured* by the propagation velocity, as *hard tissue* allows less tissue displacement than soft tissue^13^. SWE has been applied to evaluate the stiffness of various tissues, such as the gastrocnemius muscles, quadriceps muscle, peroneus longus, *tibialis anterior and patellar tendon*^11,12,14^. In addition, gray-scale ultrasound has the ability to estimate architectural parameters of the plantar fascia, such as *morphology and thickness*^15^. Our previous studies demonstrated that SWE is a valid and reliable tool for estimating the elastic properties of tendons^15^ and muscles^11^. As described, SWE provides an opportunity to quantify changes in the gastrocnemius and plantar fascia during passive ankle motion in patients with PF.

In this study, we used shear modulus values *with* SWE as an index of muscle stiffness. The aims of the present study were 1) to investigate the passive muscle stiffness of the gastrocnemius muscle in patients clinically diagnosed with PF compared with healthy controls, 2) to explore the correlation between the passive muscle stiffness and the thickness of the plantar fascia as well as the intensity of pain in patients *with PF and* 3) to detect the optimal *cut-off* points for the shear modulus of the MG and LG in identifying patients with PF.

## Methods

This study was approved by the Human Subjects Ethics Committee of the Luoyang Orthopaedic Hospital of Henan Province (approval no: KY2019-001-01). This study was conducted in accordance with the Declaration of Helsinki. The relevant guidelines and regulations of the local institute were strictly followed *during conduction. Before study initiation*, all patients were fully informed of the study purposes, experimental procedures, their rights, and the safety of ultrasonography with a statement about the experiment, and all patients signed the informed consent form.

Forty *patients* (20 asymptomatic and 20 with PF) aged between 32 and 71 years (mean age = *51.1 years* ± 12.9) participated in this study. They were recruited from Luoyang Orthopaedic Hospital of Henan Province. The inclusion criteria for the patients with PF were as follows^6,16^: 1. heel pain at the medial plantar, 2. intensity of pain upon being provoked ≥3/10 on a visual analogue scale (VAS), 3. worsening pain when waking up or after a period of rest, *4. pain* duration longer than 6 months, 5. not having neuromuscular disease, tendon rupture, musculoskeletal injury of the lower *limb or* anomaly on ultrasound. Patients who had received any treatment of the ankle or foot, *such as steroid injection, shock wave therapy and surgery, were excluded* from the study^11,16^*. For* patients with bilateral PF, the more painful leg was included in the study^11^. Twenty age-matched subjects with no history of ankle trauma or surgery or metabolic or endocrine *diseases who* did not have heel pain or inflammation were recruited as the *control group*. Furthermore, each patient was asked to wear loose-fitting short pants and to avoid longer than usual walking, standing, or running for a week prior to imaging. All patients were physically assessed by an orthopedic surgeon (S.P.) with 19 years of clinical experience and then received an SWE examination from an experienced physical therapist (Z.J.P.) *with 3 years of experience* performing ultrasonography. In addition, the SWE examination was supervised by a sonographer (S.H.S) *with 30 years of experience*.

Age, weight, height, body mass index (BMI), duration of heel pain and *the VAS score were noted*. All ultrasound examinations were performed by the ultrasound SWE scanner (Aixplorer Supersonic Imagine, France). The scanner was coupled with a 50-mm linear-array transducer (SL15-4, Supersonic Imagine, France) and used in the standard musculoskeletal mode. The upper limit (200 kPa) of the system was *adapted* for measurement of the muscle elastic modulus. Other settings of the SWE scanner were as follows: the frequency was 4~15 MHz, *the SWE options* were in penetration mode. The opacity was 85%, and the depth of the B-scan ultrasound was 3.0 cm. B-mode ultrasound was performed to assess the thickness of the plantar fascia, whereas SWE was used to assess the stiffness of the MG and LG. In the SWE examination, the Q-box diameters of the MG and LG were set as 5 × 5 mm, and the size of the regions of interest (ROIs) was 10 × 10 mm^7^.

The muscle shear modulus was measured on the painful *leg while* the ankle was passively positioned *in a relaxed position (the lowest muscle stiffness) and in neutral position (maximum tendoachilles and the plantar fascia stress)*^13^. The leg-matched healthy patients were measured as controls. Each patient was asked to lay prone on the bed with the foot fully relaxed. The upper limbs were placed on both sides of the body, and the hip and knee joints were extended. Before testing, the joint was fixed at the 90° ankle joint position using a customized and movable ankle foot orthosis. The relaxed position was what the patients perceived to be a “relaxed” foot position^13^. The angle of the ankle was measured by a manual goniometer (Sammons Preston, Royan, Canada). According to previous studies, the three measurement sites of the MG and LG were determined based on anatomical guidelines (distal, middle, and proximal regions of the muscle)^17^. *Images were obtained along the middle sagittal plane of the MG and LG at 25% (proximal region), 50% (middle region) and 75% (distal region) of the length of the muscle (toward distal tendon insertion)*^17^. All corresponding skin surfaces were marked with a black pen. To avoid the influence of muscle fatigue (if any) on the measured parameters, the passive task in the relaxed position was performed before the passive task in the *90° position*.

**Figure 1:**
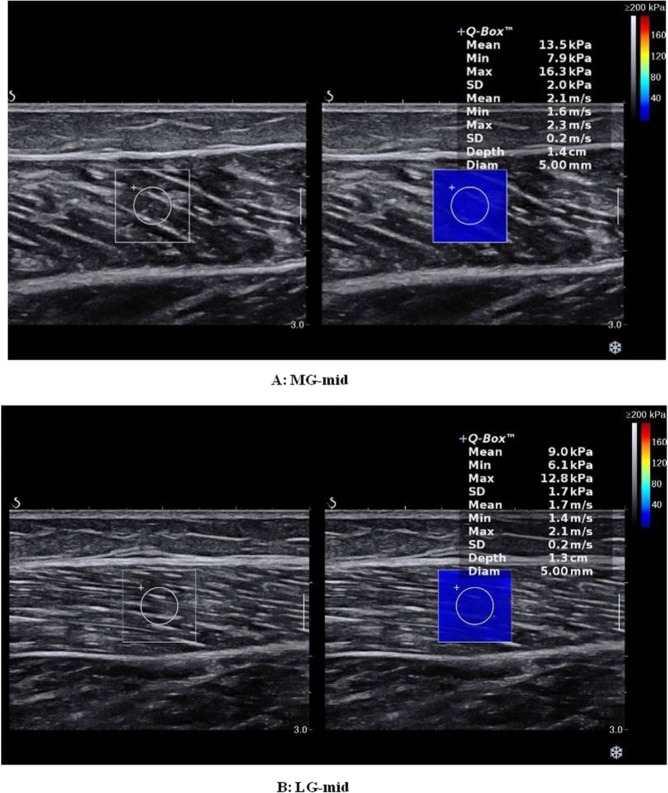
*Longitudinal* shear wave elastography scale sonograms of the MG and LG show the measurement of the shear modulus. The color-coded box indicating muscle-tendon elasticity is shown in the upper images. The longitudinal grey-scale sonograms of muscle-tendon are shown in the bottom images. The Q-Box^TM^ is shown on the right. (**A**) Shear modulus values of the MG with the ankle joint in the relaxed position. (**B**) Shear modulus values of the LG with the ankle joint the relaxed position.

As proposed previously^11^, *first*, an adequate amount of ultrasound gel was applied on the skin. The transducer was placed parallel to the muscle fibers at the marker point and positioned perpendicular to the skin. Then, *B-mode ultrasound* was activated to display the appearance of the muscle via a longitudinal section and determine the correct probe location. When the gray-scale image displayed several continuously visible fibers of the muscle, the SWE mode was turned on (Fig. 1). The transducer was kept motionless for more than 8 seconds until the color of the ROI was uniform^11^. The images were then frozen and placed in the Q-box to obtain the shear elastic modules. Three values of elastography measurements were recorded and averaged in each marker position, and a 1-minute rest was provided between each position. Care was taken not to press or deform the skin surface throughout the scanning process. All values of the shear modulus *within the Q-Box*^*TM*^ *were documented using Excel (Version 2013)*, and then mean shear modulus values were calculated.

**Figure 2:**
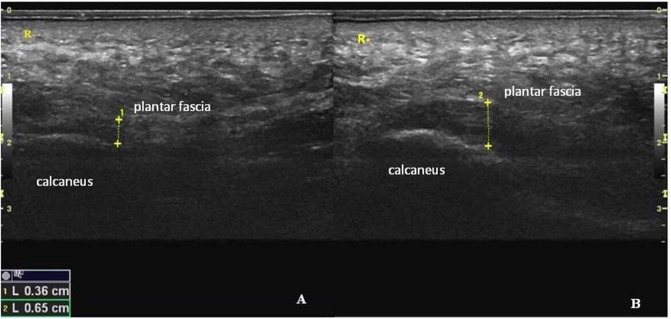
An example of a longitudinal sonogram of the plantar fascia with the corresponding thickness*. The* thickness of the plantar fascia was measured at the anterior margin of the calcaneus. (**A**) Thickness of the plantar fascia of a healthy subject. (**B**) Thickness of the plantar fascia in *patient* with PF.

The thickness of the plantar fascia was measured with a 50-mm linear-array transducer (SL15-4, Supersonic Imagine, France), and an adequate amount of ultrasound gel was applied to the plantar surface of the heel. Sagittal images were used for these ancillary tests. According to the above procedures, each patient was asked to lay prone on the bed with the foot fully relaxed. The transducer was positioned at the thickest point of the medial portion of the plantar fascia, close to the calcaneal *insertion*^15^ (Fig. 2). Then, the thickness was measured, and the image was stored for offline analyses.

The description of PF pain upon taking the first steps in the morning during daily life was evaluated with the VAS and used as an outcome measure. *The patients marked their perceived pain on a scale ranging from 0 to 10, where 0 indicates no pain and 10 indicates severe pain*.

The data were evaluated using SPSS version 17.0 (SPSS Inc, Chicago, Illinois, USA) and descriptive statistics analysis (mean and standard deviation). All data were expressed as the mean ± standard deviation. The Shapiro-Wilk test was used to test the normality of the variables. Independent *t*-tests were performed to compare the age, weight, height and BMI of the PF and the control groups. *Multivariate* analysis of covariance (MANCOVA) tests were used to compare the shear elastic modulus of the muscle (MG and LG) on the painful side in patients with PF and *the dominate side (the dominate side was defined by kicking a ball) of the controls*, with variables with significant group difference as covariates. Post hoc analysis using univariate analysis of covariance tests was performed *when the level of significance was reached*. Pearson’s correlation was used to examine the relationship between the passive muscle stiffness and the thickness of the plantar fascia as well as the intensity of pain among patients with PF. Receiver-operating characteristic (ROC) curve analysis was used to determine a *cut-off* point for the shear modulus of the MG and LG for the passive tasks that had significant group differences. The highest Youden’s index (J) represented the best overall sensitivity and specificity and was defined as the *cut-off* point for the identification of patients with and without PF. In addition, the area under the curve (AUC), the *cut-off* scores, and the sensitivity and specificity values were described. In all steps, *P values* < *0.05 were considered statistically significant*.

## Results

Among the 40 patients, 20 reported heel pain at the medial plantar and clinical diagnosis suggested the presence of PF. *The age; weight; height; BMI of the groups; the duration of heel pain and the VAS scores are summarized in* Table 1. There were no significant differences in age, weight, height, or BMI *(all P-values* > *0.05). The ICCs of the shear elastic modulus of the MG and LG muscles were 0.84 and 0.89, respectively (unpublished data on 20 subjects)*.

**Table 1:** Demographic data of subjects.

	Healthy subjects (n = 20)	Patients with PF (n = 20)	P Values
Age (years)	50.95 ± 13.08	51.25 ± 13.07	0.704
Weight (kg)	66.00 ± 10.67	69.45 ± 11.89	0.083
Height (cm)	166.55 ± 8.49	166.45 ± 8.20	0.956
Body Mass Index (kg/m^2^)	23.72 ± 2.67	24.89 ± 2.18	0.081
Duration of heel pain (months)		12.35 ± 8.03	
VAS score		4.45 ± 0.94	

Values are mean ± SD; Healthy subjects: Female 10 *(ages 36–71 years), Male 10 (a*ges 33–62 *years)*; Patients with plantar fasciitis: Female 10 *(ages 32–71 years), Male 10* (ages 35–69 *years)*. *Denotes significant difference between groups (p < 0.05).

The mean shear modulus values of the MG and LG for patients with and without PF are *summarized* in Table 2. In the relaxed position, the shear modulus values for the MG *of patients* with PF for the three regions were significantly higher than those of healthy subjects. The percent change in different regions of the MG was as follows: proximal (24.93%, *P* = 0.006), middle (25.76%, *P* = 0.000), and distal (48.90%, *P* = 0.000). No between-group differences were found for the rest of the tested regions, including the regions measured in the neutral position (all *P-*values > 0.05). In addition, the plantar fascia of patients with PF *(0.32* *cm* ± *0.08)* was significantly thicker than that of the controls *(0.26* *cm* ± *0.06*; by 23.08%; *P* = 0.022).

**Table 2:** Comparisons of shear modulus of *the MG and LG* on the painful side of patients with PF and the control subjects.

Variables Shear elastic modulus (kPa)			Healthy group	PF group	P values
Relaxation position	MG	Proximal	26.23 ± 6.65	32.77 ± 7.41	0.006*
		Middle	13.12 ± 2.80	16.50 ± 1.66	0.000*
		Distal	15.01 ± 3.67	22.35 ± 6.78	0.000*
	LG	Proximal	26.10 ± 9.95	29.20 ± 10.95	0.353
		Middle	11.45 ± 2.18	12.09 ± 1.56	0.288
		Distal	18.48 ± 9.49	20.75 ± 7.38	0.404
Neutral position (90°)	MG	Proximal	49.06 ± 6.80	54.41 ± 12.48	0.100
		Middle	38.63 ± 9.09	43.32 ± 10.49	0.139
		Distal	45.22 ± 7.91	50.25 ± 16.53	0.228
	LG	Proximal	45.38 ± 7.16	47.24 ± 13.83	0.596
		Middle	33.11 ± 7.16	34.10 ± 8.98	0.702
		Distal	37.92 ± 6.68	37.27 ± 11.84	0.831

MG: Medial head of the gastrocnemius muscle; LG: Lateral head of the gastrocnemius muscle. *Denotes significant difference between groups (p < 0.05).

The relationship between the shear modulus of patients with PF and the intensity of pain are *summarized* in Table 3. When the ankle was in the relaxation position, significant correlations were found between pain and the shear modulus of the middle and distal region of the MG *(r* = *0.509, P* = *0.022; r* = *0.521, P* = *0.018)*. No significant correlation was found between pain and the rest of the tested regions, including all regions measured in the neutral position *(all P-values* > *0.05)*. A correlation analysis for secondary variables was also carried out. No correlation was observed between the intensity of pain and the thickness of the plantar fascia (*r* = 0.193, *P* = 0.416).

**Table 3:** *Correlation* between shear modulus of patients with PF and pain score.

***Variables***			***Shear modulus of Patients with PF (kPa)***	***r***	***p***
*Relaxation position*	*MG*	*Proximal*	*32.77* ± *7.41*	*0.142*	*0.551*
		*Middle*	*16.50* ± *1.66*	*0.509*	0.022*
		*Distal*	*22.35* ± *6.78*	*0.521*	0.018*
	*LG*	*Proximal*	*29.20* ± *10.95*	*0.175*	*0.461*
		*Middle*	*12.79* ± *2.08*	*0.412*	*0.071*
		*Distal*	*20.75* ± *7.38*	*0.022*	*0.928*
*Neutral position*	*MG*	*Proximal*	*54.41* ± *12.48*	*0.002*	*0.993*
		*Middle*	*43.32* ± *10.49*	*0.099*	*0.678*
		*Distal*	*50.25* ± *16.53*	*0.016*	*0.945*
	*LG*	*Proximal*	*45.24* ± *15.45*	*0.161*	*0.498*
		*Middle*	*33.60* ± *9.57*	*0.119*	*0.618*
		*Distal*	*37.27* ± *11.84*	*0.251*	*0.285*
*Plantar fascia*		*Thickness*	*0.32* ± *0.08*	*0.193*	*0.416*

*Pain score of patients with PF is 4.45* ± *0.94*.

*Denotes significant correlation (p < 0.05).

ROC curves were constructed to determine the optimal cut-off point for the shear modulus of the MG and LG measured while the ankle was in the relaxed position and in the neutral position for identifying patients with PF. When considering the ankle in the relaxed position, *the AUC of the MG was 0.749 (P* = *0.007) in the proximal region, 0.844 (P* = *0.000) in the middle region and 0.893 (P* = *0.000) in the distal region. Youden’s index showed that when the ankle was in the relaxed position, a shear modulus of 29.08 kPa for the MG in the proximal region had a sensitivity of 0.75 and specificity of 0.25, a shear modulus of 14.60 kPa in the middle region had a sensitivity of 0.90 and specificity of 0.25, and a shear modulus of 18.0 kPa in the distal region had a sensitivity of 0.85 and a specificity of 0.20 for identifying patients at risk for PF*. The AUCs for the rest of the tested regions were not statistically significant *and no cut-off* point was found. The AUCs for other measuring points were not statistically significant, and no *cut-off p*oint was found *(all P-values* > *0.05)*.

## Discussion

By taking advantage of SWE, the present study showed that patients with PF exhibited greater muscle stiffness when the ankle was in a relaxed *position. The* increase was observed only in the MG (proximal region, middle region, and distal region), but *not in* the neutral position. More importantly, significant correlations *were found between* pain and the shear modulus of MG (the middle and distal region) when the ankle was in a relaxed position. In addition, we determined the *initial* cut-off points for the shear modulus of the MG when the ankle was in a relaxed position, and they can be used for the identification of patients with PF.

*Interestingly, increased muscle stiffness was detected in the MG but not in the LG in patients with PF when the ankle joint was relaxed. The passive muscle stiffness of the healthy group and the PF group was not significantly different when the ankle was in the neutral position. This is the first study to find a change in the passive muscle stiffness of the gastrocnemius in patients with PF compared with healthy controls. Most previous studies have demonstrated differences between the MG and LG in various human activities; the proportion of MG is larger than that of LG*^12,18–20^. For example, the impact on the MG and LG during walking and running might be different among patients. *Green et al*. found that exercise-induced muscle stiffness was different in the muscle heads in the *calf and* that the effect of exercise on muscle stiffness was specific to muscle heads within a muscle group^12^. *Stenroth et al*. found that a longer distance walked in a 6-minute walk test was significantly associated with MG^18^. In addition, *Hirata et al*. reported that passive muscle stiffness differs among the triceps surae. In their study, the elastic modulus of the MG and LG was quantified using SWE^19^. They also found a higher elastic modulus for the MG than for the LG. After static stretching, a significant reduction in the elastic modulus of the MG but *not for* the LG was observed^19^. Furthermore, *Masood et al*. reported a significant difference in the activity of the MG and LG^20^. Electromyography (EMG) was used to assess the activity of the MG and LG. The authors found an increase in the activity of the MG (34%) and LG (21%) during sustained submaximal isometric exercise^20^. *All the above studies demonstrate that the MG experiences greater mechanical loading during daily activities*. Clinically, most studies have reported that reduced muscle stiffness can alleviate the symptoms of patients with PF^2,6,9,21^. For example, chronic symptoms lasting longer than 4 months might cause the tissue to be stiffer and to become more difficult to stretch^2^. *Stretching* of the gastrocnemius-AT complex is widely considered to be an effective treatment for PF and reducing foot and ankle pain because stretching addresses PF caused by tight gastrocnemius and intrinsic foot muscles^2,6,9^. After eccentric training, the fascicle length of the MG increased 12%, but this change was *not observed for* the LG. There was a greater response to eccentric training by the MG than the LG^21^. In addition, *during manual therapy*, the most frequently used modalities were massage, specific soft-tissue *mobilization and* myofascial release^10^. All of these interventions were effective for PF, as they reduced the stiffness of the muscles and tendon^10^. In summary, increased muscle stiffness is not similar for the MG and LG. This finding may provide evidence for new treatment modalities, and current clinical treatment programs for PF-related diseases *may be* tailored to the MG to treat patients with PF.

The greater muscle stiffness of patients with PF measured with the ankle in the relaxed position is likely explained by the intrinsic changes in muscle mechanical properties. One of the reasons may lie in the anatomy of the MG and LG. First, both the MG and LG are *plantar flexors* but contribute to different degrees, with the MG providing more than 70% of the muscle force^22^. Second, the MG is longer and larger and extends more distally in the calf than the LG^23^, and the muscle volume of the MG is greater than that of the LG^24^. Third, a study reported that the AT has a unique twisted descending structure, which enables it to handle the functional loads applied to the tendon^25^*. Pekala et al*. found that the torsion of the MG was significantly lower than that of the LG *and that* the rotation angle of the LG is approximately 5 times that of the MG. *The fibers originating from the LG rotate an average of 135.98°* ± *33.58, while those of the MG twist 28.17°* ± *15.1526. The footprint of the fascicles of the MG tendon forms the superficial part of the AT, which is inserted over the entire width of the calcaneal tuberosity. This anatomical structure suggests that this is the part of the MG tendon that is connected with the plantar fascia. The fascicles from the LG tendon insert on the lateral aspect of the middle facet of the calcaneal tuberosi*ty^7^. In all of the above views of biomechanics, *the variability in the plantar fascia is closely related to the MG*. The contribution of the MG to the plantar fascia is larger than that of the *LG. This* increased muscle *tension has* clinical consequences. Biomechanically, the transmission and generation of triceps surae muscle forces *are* affected by the compliance of the AT^23^. *Thus, the increased tension with PF may decrease the degree of activation and result in prolonged onset activation of muscles and tendons and altered ankle kinematics. These are relevant to a greater superior translation of the ankle joint with low efficiency in plantar flexion and stabilization of the ankl*e^3,8,22,26^. *The end result is that these changes may compromise the plantar fascia and contribute to PF*^6^. However, the passive muscle stiffness was not significantly different when the ankle was in the neutral position, which is likely explained by the similar passive stiffness produced by the muscles in patients with or without PF. *Further study are required to explain the differences in the ankle angle of patients with and without PF, as well as the change in muscle stiffness in patients with PF before and after treatment (e.g., stretching, massage)*.

*Our study demonstrated that the passive stiffness differs among the three areas within each muscle, with the smallest stiffness being in the middle area and the highest in the proximal area. Biomechanically, the most likely explanation is that the gastrocnemius muscle (MG and LG) has a slack connection and low compliance between muscle bellies, which leaves a margin for heterogeneous non-uniform behavior during low levels of force generation or in the passive state*^25,27^*. Once in a more active state, the stiffness of muscle connections leads to more uniform behavior*^25,27^*. Therefore, the smallest stiffness is found in the middle area. This is also why greater stiffness of the MG is found in the relaxed position, not in the neutral position. In addition, due to the anatomic structure of the MG and LG*^24^*, the proximal portions of the gastrocnemius fascicles insert at the femoral condyle level, which presents as curvilinear directions. Muscle fascicles that are not compliant increase muscle stiffness*^14,17^*. The distal portions of the gastrocnemius fascicles are directly attached to the tendon*^28^*. Moreover, the proximal and distal portions are near aponeuroses, which may also result in increased stiffness*^14,17^*. Overall, the smallest stiffness of the MG and LG was observed in the middle area, and the highest stiffness was in the proximal area*.

Our study also demonstrated a significant *correlation between* the stiffness of the MG and the intensity of pain in individuals with PF. Greater stiffness of the MG may indicate more pain in individuals with PF. Note that the MG connects with the plantar fascia by means of the AT^8,26^. Given this anatomic relationship, *the stiffness of the plantar fascia is closely related to the MG. Stiffness in the MG may induce stiffness in the plantar fascia*^8^. I*n a cadaver study, the strain of the plantar fascia increased with increasing calf muscle stiffness*^8^. *Findings from the study have demonstrated a close relationship between the stiffness of the MG and pain in patients with PF. More importantly, the findings suggest that releasing MG stiffness is recommended in the treatment of patients with PF*.

We also found that the plantar fascia of patients with PF was significantly thicker than that in healthy subjects, *and the thickness of the plantar fascia was not associated with pain. This is consistent with previous findings. Chen et al. performed a laboratory-based study to analyze whether the thickness of the plantar fascia was related to pain and foot dysfunction in patients with chronic unilateral PF*^29^. *They found that patients with PF had significantly thicker fascia on the painful foot compared with the healthy group*. A direct correlation was observed between the thickness of the plantar fascia and function but not pain in PF^29^. In addition, *Maki et al. also found that the thickness of the plantar fascia was significantly different between a PF group and a healthy group*^30^. *On magnetic resonance imaging (MRI) findings, the mean thickness of the plantar fascia of healthy feet was 3.1* *mm* ± *1.0, while it was 4.4* *mm* ± *1.6 for the affected feet*. Furthermore, *Gamba et al. used ultrasound and MRI to identify a correlation between the thickness of plantar fascia and pain*^31^. *They found that the thickness of the plantar fascia in patients with PF did not correlate with pain using a VAS*^31^*. The findings from this study probably reflect a relationship that does not correlate with the thickness of the plantar fascia and pain*.

*As discussed above, an increase in the passive shear modulus of muscle may be due to an overload of the plantar fascia related to sports participation. Preventive instruments such as stretching of the plantar fascia and muscles as well as silicone insoles are essential. Given the high prevalence of PF in the general population, early identification of individuals at risk for PF is extremely important*. For this reason, we found *an initial cut-off stiffness* of the MG (29.08 kPa, sensitivity = 0.75) when the ankle was in a relaxed position for identifying patients with PF. In other words, patients with a passive stiffness of the MG greater than 29.08 kPa may have a higher risk of developing PF.

The present study has some limitations. First, EMG was not used during the tests to monitor the muscle activity and ensure the muscles were not contracting. However, all patients were verbally instructed to stay relaxed, and *no signs of muscle contraction were observed on the gray-scale image. Based on this, we are confident that patients or subjects remained in a passive state during passive dorsiflexion*. Second, because of the limitation of SWE, the shear modulus of the soleus muscle and plantar fascia was not obtained in this study, but we investigated three regions within the MG and LG. *Third, the structure and size of the muscles were not evaluated in this study. The force-generating capacity of the muscles is dependent on their physiological cross-sectional area and pennation angle. Although the stiffness is not affected by the anatomical cross-sectional area and pennation angle, further studies need to investigate the effects of these parameters on the gastrocnemius in patients with and without PF*.

## Conclusions

Patients with PF have increased passive muscle stiffness in the MG but not in the LG when the ankle joint is in a relaxed *position. In* addition, *an increase in passive stiffness of the MG is associated with pain*. The initial *cut-off* points of the muscles’ shear modulus with the ankle in a resting position need to be determined so that they can be used for the identification of patients with PF. *These findings indicate that the stiffness of muscles is a good indicator for patients with PF in clinical practice. A muscle-specific approach is recommended for the prevention of PF and the treatment of patients with PF. We suggest that the stiffness of muscles should be used to evaluate PF and that treatment programs for PF diseases be tailored to the MG. Traditional treatment of the whole gastrocnemius muscle might not be targeted at the tight muscle*.

### Practical implications

- Patients with PF have increased passive muscle stiffness in the MG but not in the LG.
- *An increase in the passive stiffness of the MG is associated with the intensity of pain in patients with PF*.
- Shear wave elastography measurement of gastrocnemius stiffness can identify patients at risk for PF.
- Releasing and stretching muscle programs should focus on the MG for prevention and rehabilitation in patients with PF.

## Data Availability

All data included in this study are available upon request by contact with the corresponding author.
